# 12-(4-Meth­oxy­benzo­yl)-2-methyl­benzo[*f*]pyrido[1,2-*a*]indole-6,11-dione

**DOI:** 10.1107/S1600536812040408

**Published:** 2012-09-29

**Authors:** J. Josephine Novina, G. Vasuki, Yun Liu, Jin-Wei Sun

**Affiliations:** a Department of Physics, Idhaya College for Women, Kumbakonam-1, India; b Department of Physics, Kunthavai Naachiar Govt. Arts College (W) (Autonomous), Thanjavur-7, India; c Institute of Chemistry and Chemical Engineering, Xuzhou Normal University, Xuzhou 221116, Jiangsu, People’s Republic of China

## Abstract

In the title compound, C_25_H_17_NO_4_, the indolizine fused naphthaquinone unit is approximately planar [r.m.s deviation = 0.0678 Å] and makes a dihedral angle of 57.82 (5)° with the benzene ring of the meth­oxy­benzene group. The naphtho­quinone O atoms deviate, in the same sense, from the mean plane of the fused six-membered rings by 0.2001 (14) and 0.0516 (14) Å. In the crystal there is π–π stacking of inversion-related pairs of mol­ecules [inter­planar spacing = 3.514 (2) Å].

## Related literature

For general background to the applications and biological activity of indolizine derivatives, see: Švorc *et al.* (2009 ▶). For the synthesis of indolizines, see: Babaev *et al.* (2005 ▶), and for their use as inter­mediates in the synthesis of indolizidines, see: Kloubert *et al.* (2012 ▶). For the crystal structures of similar compounds, see: Liu *et al.* (2011 ▶); Ramesh *et al.* (2009 ▶). For standard bond lengths, see: Allen *et al.* (1987 ▶).
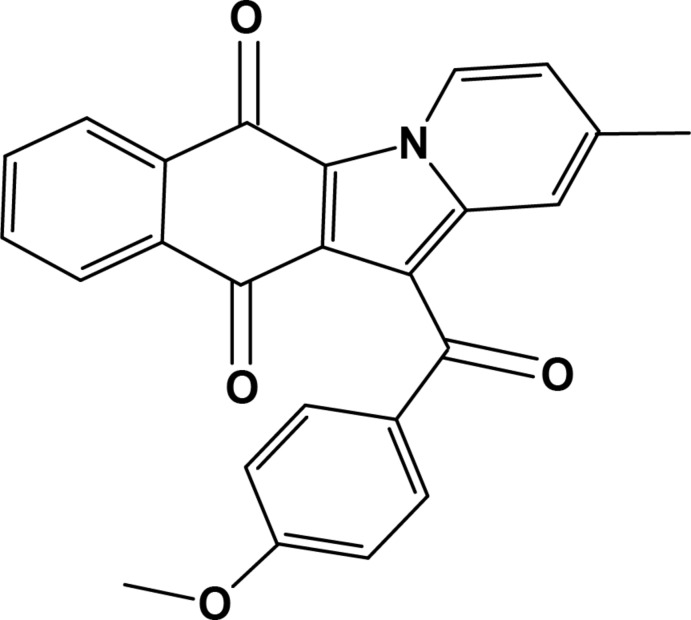


## Experimental

### 

#### Crystal data

C_25_H_17_NO_4_
*M* *_r_* = 395.40
Monoclinic, 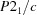

*a* = 8.1346 (3) Å
*b* = 23.2926 (8) Å
*c* = 10.1505 (3) Å
β = 97.304 (2)°
*V* = 1907.67 (11) Å^3^
*Z* = 4
Mo *K*α radiation
μ = 0.09 mm^−1^
*T* = 293 K
0.30 × 0.20 × 0.20 mm

#### Data collection

Bruker Kappa APEXII CCD diffractometer
Absorption correction: multi-scan (*SADABS*; Bruker, 2004 ▶) *T*_min_ = 0.972, *T*_max_ = 0.982
18096 measured reflections
3852 independent reflections
2856 reflections with *I* > 2σ(*I*)
*R*_int_ = 0.029

#### Refinement

*R*[*F* ^2^ > 2σ(*F* ^2^)] = 0.043
*wR*(*F* ^2^) = 0.120
*S* = 1.04
3852 reflections
271 parameters
H-atom parameters constrained
Δρ_max_ = 0.26 e Å^−3^
Δρ_min_ = −0.20 e Å^−3^

### 

Data collection: *APEX2* (Bruker, 2004 ▶); cell refinement: *APEX2* and *SAINT* (Bruker, 2004 ▶); data reduction: *SAINT* and *XPREP* (Bruker, 2004 ▶); program(s) used to solve structure: *SHELXS97* (Sheldrick, 2008 ▶); program(s) used to refine structure: *SHELXL97* (Sheldrick, 2008 ▶); molecular graphics: *ORTEP-3 for Windows* (Farrugia, 1997 ▶); software used to prepare material for publication: *PLATON* (Spek, 2009 ▶).

## Supplementary Material

Crystal structure: contains datablock(s) I, global. DOI: 10.1107/S1600536812040408/pk2443sup1.cif

Structure factors: contains datablock(s) I. DOI: 10.1107/S1600536812040408/pk2443Isup2.hkl

Supplementary material file. DOI: 10.1107/S1600536812040408/pk2443Isup3.cml

Additional supplementary materials:  crystallographic information; 3D view; checkCIF report

## Figures and Tables

**Fig. 1.**
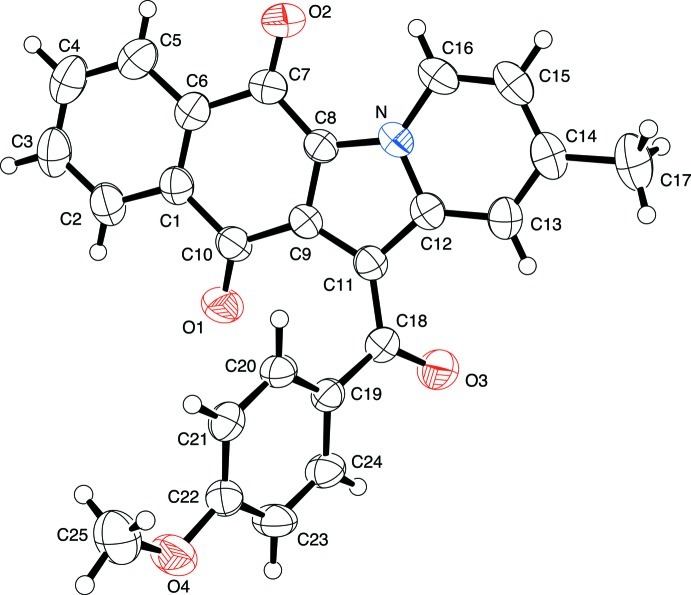
The molecular structure of the title compound with displacement ellipsoids drawn at the 50% probability level.

**Fig. 2.**
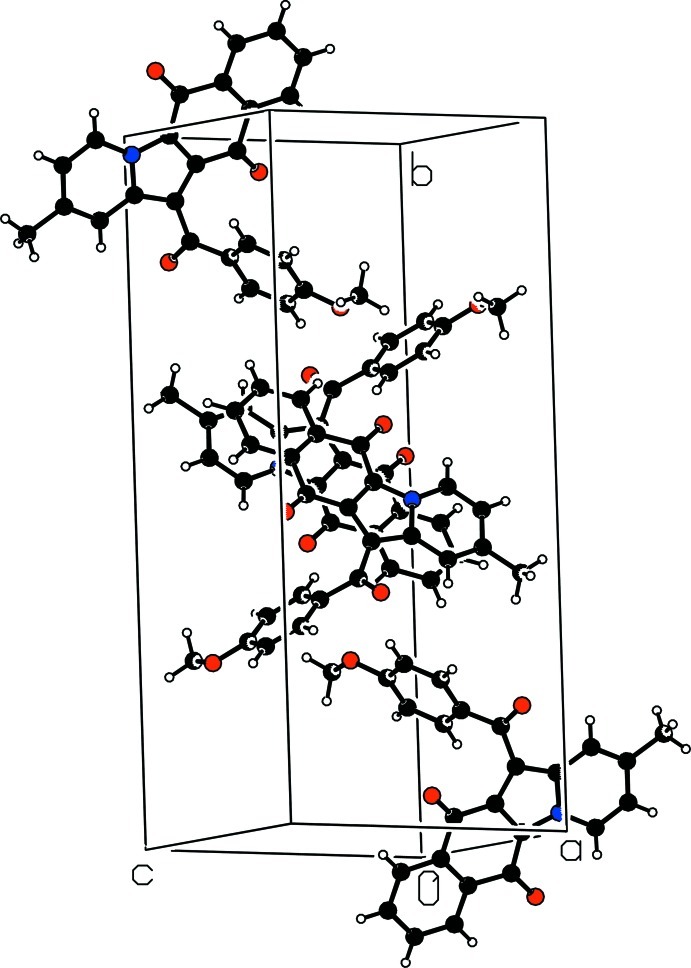
Crystal packing of the title compound viewed approximately down the bisector of the a and c axes.

## supplementary crystallographic information

### Comment

Indolizines, the nitrogen containing heterocyclic systems, are widely distributed in nature. In particular, indolizine derivatives are an important class of heterocyclic bioactive compounds with a wide range of applications, such as pharmaceutical drugs, potential central nervous system depressants, calcium entry blockers, cardiovascular agents, spectral sensitizers and novel dyes. Polycyclic indolizine derivatives have been found to have high-efficiency long-wavelength fluorescence quantum yield. Several polyhydroxylated indolizines are interesting as inhibitors of glycosides. They have also been tested as antimycobacterial agents against mycobacterial tuberculosis, for the treatment of angina pectoris, aromatase inhibitory, antiinflammatory, antiviral, analgesic and antitumor activities (Švorc *et al.*, 2009). Moreover, the application of indolizines themselves are as intermediates in the synthesis of indolizidines (Kloubert *et al.*, 2012) and many natural alkaloids contain in their structure a saturated (swainsonine) or aromatic (camptothecin) indolizine moiety (Babaev *et al.*, 2005). The benzo[*f*]pyrido[1,2-*a*]indole-6,11-diones are benzo-fused indolizines, and occur in several marine alkaloids (Liu *et al.*, 2011). The synthesis of these compounds has drawn much research interest. In view of their importance, the crystal structure determination of the title compound was carried out and results are presented herein.

In the title compound, C_25_H_17_NO_4_, the fused naphthaquione–indolizine ring system (N/C1–C16/O1/O2) is approximately planar with a maximum deviation of 0.1193 (14) Å for atom C11 and -0.2001 (14) Å for atom O1, respectively. The fused ring systems make a dihedral angle of 57.82 (5)° with that of benzene ring of the methoxybenzene group. The torsion angles C11—C18—C19—C20 = -21.0 (2)° and C11—C18—C19—C24 = 161.52 (16)° also indicate that the aromatic ring is at different plane from the plane of the fused ring systems. The sum of bond angles around N [359.99 (43)°] indicates that atom N exhibits sp^2^ hybridization. The geometric parameters of the title compound (Fig. 1) agree well with a reported similar structure 12-benzoyl-2-methylnaphtho[2,3-*b*]-indolizine-6,11-dione [Liu *et al.*, 2011]. The O2 atom is essentially coplanar with the ring, deviating by only -0.0516 (14) Å, while O1 deviates by -0.2001 (14) Å from the best-fit plane. The discrepancy in bond length is also observed for C9—C11 [1.400 (2) Å], which is slightly shorter than the average of 1.434 (1) Å calculated for indoles in the Cambridge Structure Database (Allen *et al.*, 1987).

The endocyclic angle at C7 is contracted to 114.76 (15)° while those at C8 is expanded to 125.71 (15)°, respectively. This would appear to be a real effect caused by the fusion of the indolizine with naphthalene ring resulting an angular distortion as observed in the reported structure 3'-benzyloxy-3-hydroxy-3,3'-bi-1*H*-indole-2,2'(3*H*,3'H)-dione monohydrate [Ramesh *et al.*, 2009]. The widening of exocyclic angle O4—C22—C21 [125.05 (16)°] from the normal value of 120°, may be due to steric repulsion between atoms H21 and H25C (H21—H25C = 2.367 Å). In the crystal, there is π-π stacking of inversion-related pairs of molecules [interplanar spacing = 3.514 (2) Å].

### Experimental

4-Methyl pyridine (3.0 mmol), 2-bromo-1-(4-methoxyphenyl)ethanone (1.0 mmol), 1,4-naphthaquionone (1.0 mmol), and hydrated copper chloride (0.1 mmol) were mixed in 15 ml of CH_3_CN and heated to reflux for 12 h. After completion of the reaction, the reaction mixture was separated by silica gel column chromatography to afford the title compound (yield: 91%).

### Refinement

All H atoms were positioned geometrically and treated as riding on their parent atoms: C—H =0.93 and 0.96 Å for CH and CH_3_ H atoms, respectively, with *U*_iso_(H) =K*U*_eq_ (parent C-atom), where K=1.5 for CH_3_ H atoms and K=1.2 for CH H-atoms.

### Crystal data

**Table:**

C_25_H_17_NO_4_	*F*(000) = 824
*M**_r_* = 395.40	*D*_x_ = 1.377 Mg m^−^^3^
Monoclinic, *P*2_1_/*c*	Mo *K*α radiation, λ = 0.71073 Å
Hall symbol: -P 2ybc	Cell parameters from 5015 reflections
*a* = 8.1346 (3) Å	θ = 2.2–26.3°
*b* = 23.2926 (8) Å	µ = 0.09 mm^−^^1^
*c* = 10.1505 (3) Å	*T* = 293 K
β = 97.304 (2)°	Block, brown
*V* = 1907.67 (11) Å^3^	0.30 × 0.20 × 0.20 mm
*Z* = 4	

### Data collection

**Table:**

Bruker Kappa APEXII CCD diffractometer	3852 independent reflections
Radiation source: fine-focus sealed tube	2856 reflections with *I* > 2σ(*I*)
Graphite monochromator	*R*_int_ = 0.029
ω and φ scan	θ_max_ = 26.3°, θ_min_ = 2.2°
Absorption correction: multi-scan (*SADABS*; Bruker, 2004)	*h* = −10→10
*T*_min_ = 0.972, *T*_max_ = 0.982	*k* = −28→29
18096 measured reflections	*l* = −12→10

### Refinement

**Table:**

Refinement on *F*^2^	Primary atom site location: structure-invariant direct methods
Least-squares matrix: full	Secondary atom site location: difference Fourier map
*R*[*F*^2^ > 2σ(*F*^2^)] = 0.043	Hydrogen site location: inferred from neighbouring sites
*wR*(*F*^2^) = 0.120	H-atom parameters constrained
*S* = 1.04	*w* = 1/[σ^2^(*F*_o_^2^) + (0.0533*P*)^2^ + 0.4967*P*] where *P* = (*F*_o_^2^ + 2*F*_c_^2^)/3
3852 reflections	(Δ/σ)_max_ < 0.001
271 parameters	Δρ_max_ = 0.26 e Å^−^^3^
0 restraints	Δρ_min_ = −0.20 e Å^−^^3^

### Special details

**Table:**

Geometry. All e.s.d.'s (except the e.s.d. in the dihedral angle between two l.s. planes) are estimated using the full covariance matrix. The cell e.s.d.'s are taken into account individually in the estimation of e.s.d.'s in distances, angles and torsion angles; correlations between e.s.d.'s in cell parameters are only used when they are defined by crystal symmetry. An approximate (isotropic) treatment of cell e.s.d.'s is used for estimating e.s.d.'s involving l.s. planes.
Refinement. Refinement of *F*^2^ against ALL reflections. The weighted *R*-factor *wR* and goodness of fit *S* are based on *F*^2^, conventional *R*-factors *R* are based on *F*, with *F* set to zero for negative *F*^2^. The threshold expression of *F*^2^ > σ(*F*^2^) is used only for calculating *R*-factors(gt) *etc*. and is not relevant to the choice of reflections for refinement. *R*-factors based on *F*^2^ are statistically about twice as large as those based on *F*, and *R*- factors based on ALL data will be even larger.

### Fractional atomic coordinates and isotropic or equivalent isotropic displacement parameters (Å^2^)

**Table:**

	*x*	*y*	*z*	*U*_iso_*/*U*_eq_	
N	0.17994 (16)	0.53308 (6)	0.57068 (13)	0.0388 (3)	
O2	0.34773 (17)	0.42161 (6)	0.56349 (14)	0.0628 (4)	
C8	0.24197 (19)	0.50723 (7)	0.46488 (15)	0.0376 (4)	
C9	0.21764 (19)	0.54500 (7)	0.35825 (16)	0.0383 (4)	
O1	0.2104 (2)	0.55356 (6)	0.12630 (12)	0.0652 (4)	
C6	0.3785 (2)	0.43608 (7)	0.33619 (18)	0.0435 (4)	
C18	0.0992 (2)	0.64903 (7)	0.32447 (17)	0.0439 (4)	
C19	0.2224 (2)	0.67504 (7)	0.24748 (16)	0.0379 (4)	
C1	0.3460 (2)	0.47115 (7)	0.22307 (18)	0.0443 (4)	
C21	0.5041 (2)	0.68874 (7)	0.20229 (17)	0.0431 (4)	
H21	0.6165	0.6806	0.2219	0.052*	
O3	−0.03169 (17)	0.67344 (7)	0.33481 (16)	0.0714 (5)	
C16	0.1778 (2)	0.51365 (9)	0.69868 (16)	0.0477 (4)	
H16	0.2195	0.4776	0.7238	0.057*	
C10	0.2554 (2)	0.52654 (7)	0.22716 (17)	0.0444 (4)	
C12	0.11594 (19)	0.58666 (7)	0.53067 (16)	0.0399 (4)	
C7	0.3231 (2)	0.45262 (8)	0.46517 (17)	0.0426 (4)	
C14	0.0491 (2)	0.60280 (9)	0.75177 (18)	0.0509 (5)	
C23	0.2821 (2)	0.74026 (8)	0.0772 (2)	0.0539 (5)	
H23	0.2453	0.7662	0.0101	0.065*	
O4	0.54786 (16)	0.75454 (6)	0.02430 (14)	0.0634 (4)	
C20	0.3900 (2)	0.66254 (7)	0.27206 (16)	0.0409 (4)	
H20	0.4265	0.6358	0.3373	0.049*	
C13	0.0496 (2)	0.62101 (8)	0.62437 (18)	0.0464 (4)	
H13	0.0052	0.6567	0.5991	0.056*	
C22	0.4494 (2)	0.72723 (7)	0.10299 (17)	0.0443 (4)	
C2	0.3967 (2)	0.45322 (9)	0.1042 (2)	0.0553 (5)	
H2	0.3732	0.4758	0.0285	0.066*	
C11	0.14110 (19)	0.59490 (7)	0.39734 (16)	0.0403 (4)	
C24	0.1712 (2)	0.71529 (8)	0.14967 (18)	0.0483 (4)	
H24	0.0598	0.7253	0.1334	0.058*	
C15	0.1142 (2)	0.54769 (9)	0.78676 (18)	0.0539 (5)	
H15	0.1129	0.5346	0.8732	0.065*	
C4	0.5135 (3)	0.36798 (9)	0.2080 (2)	0.0644 (6)	
H4	0.5702	0.3335	0.2030	0.077*	
C5	0.4614 (2)	0.38467 (8)	0.3266 (2)	0.0538 (5)	
H5	0.4824	0.3611	0.4008	0.065*	
C17	−0.0160 (3)	0.63983 (11)	0.8546 (2)	0.0731 (7)	
H17A	−0.0058	0.6197	0.9379	0.110*	
H17B	−0.1305	0.6486	0.8270	0.110*	
H17C	0.0467	0.6748	0.8649	0.110*	
C3	0.4816 (3)	0.40227 (9)	0.0974 (2)	0.0646 (6)	
H3	0.5174	0.3911	0.0178	0.077*	
C25	0.7210 (2)	0.74482 (11)	0.0475 (3)	0.0754 (7)	
H25A	0.7748	0.7666	−0.0149	0.113*	
H25B	0.7430	0.7047	0.0368	0.113*	
H25C	0.7624	0.7565	0.1362	0.113*	

### Atomic displacement parameters (Å^2^)

**Table:**

	*U*^11^	*U*^22^	*U*^33^	*U*^12^	*U*^13^	*U*^23^
N	0.0378 (7)	0.0458 (8)	0.0328 (7)	−0.0053 (6)	0.0042 (6)	0.0017 (6)
O2	0.0696 (9)	0.0613 (9)	0.0577 (8)	0.0171 (7)	0.0086 (7)	0.0195 (7)
C8	0.0367 (8)	0.0409 (9)	0.0352 (9)	−0.0036 (7)	0.0045 (7)	0.0012 (7)
C9	0.0375 (8)	0.0406 (9)	0.0370 (9)	−0.0022 (7)	0.0061 (7)	0.0005 (7)
O1	0.1040 (11)	0.0559 (8)	0.0363 (7)	0.0112 (8)	0.0117 (7)	0.0047 (6)
C6	0.0366 (9)	0.0411 (10)	0.0529 (11)	−0.0042 (7)	0.0058 (8)	−0.0034 (8)
C18	0.0452 (10)	0.0427 (10)	0.0444 (10)	0.0067 (8)	0.0081 (8)	0.0021 (8)
C19	0.0417 (9)	0.0344 (9)	0.0377 (9)	0.0031 (7)	0.0049 (7)	−0.0014 (7)
C1	0.0436 (9)	0.0431 (10)	0.0479 (10)	−0.0067 (7)	0.0123 (8)	−0.0052 (8)
C21	0.0389 (9)	0.0444 (10)	0.0453 (10)	0.0049 (7)	0.0033 (7)	−0.0012 (8)
O3	0.0561 (8)	0.0741 (10)	0.0893 (11)	0.0229 (7)	0.0302 (8)	0.0266 (8)
C16	0.0476 (10)	0.0604 (12)	0.0346 (9)	−0.0068 (9)	0.0031 (8)	0.0086 (9)
C10	0.0543 (10)	0.0411 (10)	0.0388 (10)	−0.0048 (8)	0.0097 (8)	0.0002 (8)
C12	0.0353 (8)	0.0445 (9)	0.0402 (9)	−0.0055 (7)	0.0056 (7)	−0.0012 (8)
C7	0.0374 (9)	0.0446 (10)	0.0447 (10)	−0.0034 (7)	0.0014 (7)	0.0053 (8)
C14	0.0436 (10)	0.0670 (13)	0.0433 (10)	−0.0125 (9)	0.0095 (8)	−0.0108 (9)
C23	0.0517 (11)	0.0515 (11)	0.0579 (12)	0.0064 (9)	0.0044 (9)	0.0211 (9)
O4	0.0549 (8)	0.0670 (9)	0.0710 (9)	−0.0051 (7)	0.0179 (7)	0.0200 (7)
C20	0.0460 (9)	0.0394 (9)	0.0365 (9)	0.0073 (7)	0.0017 (7)	0.0036 (7)
C13	0.0422 (9)	0.0507 (10)	0.0473 (10)	−0.0040 (8)	0.0102 (8)	−0.0081 (8)
C22	0.0485 (10)	0.0394 (9)	0.0462 (10)	−0.0028 (8)	0.0108 (8)	−0.0006 (8)
C2	0.0608 (12)	0.0542 (11)	0.0543 (11)	−0.0064 (9)	0.0202 (9)	−0.0091 (9)
C11	0.0399 (9)	0.0423 (9)	0.0395 (9)	−0.0012 (7)	0.0079 (7)	0.0011 (7)
C24	0.0383 (9)	0.0473 (10)	0.0582 (11)	0.0071 (8)	0.0024 (8)	0.0112 (9)
C15	0.0534 (11)	0.0751 (14)	0.0335 (9)	−0.0127 (10)	0.0073 (8)	−0.0006 (9)
C4	0.0555 (12)	0.0530 (12)	0.0865 (16)	0.0040 (10)	0.0153 (11)	−0.0176 (12)
C5	0.0478 (10)	0.0449 (10)	0.0682 (13)	0.0010 (8)	0.0054 (9)	−0.0037 (9)
C17	0.0779 (15)	0.0912 (17)	0.0537 (12)	−0.0094 (13)	0.0219 (11)	−0.0256 (12)
C3	0.0655 (13)	0.0615 (13)	0.0712 (14)	−0.0054 (11)	0.0266 (11)	−0.0222 (12)
C25	0.0506 (12)	0.0877 (17)	0.0921 (17)	−0.0101 (11)	0.0251 (12)	0.0067 (14)

### Geometric parameters (Å, º)

**Table:**

N—C16	1.378 (2)	C14—C13	1.361 (3)
N—C8	1.381 (2)	C14—C15	1.416 (3)
N—C12	1.393 (2)	C14—C17	1.502 (3)
O2—C7	1.228 (2)	C23—C24	1.365 (2)
C8—C9	1.389 (2)	C23—C22	1.386 (3)
C8—C7	1.433 (2)	C23—H23	0.9300
C9—C11	1.400 (2)	O4—C22	1.359 (2)
C9—C10	1.467 (2)	O4—C25	1.416 (2)
O1—C10	1.218 (2)	C20—H20	0.9300
C6—C5	1.384 (2)	C13—H13	0.9300
C6—C1	1.407 (2)	C2—C3	1.379 (3)
C6—C7	1.489 (2)	C2—H2	0.9300
C18—O3	1.223 (2)	C24—H24	0.9300
C18—C19	1.477 (2)	C15—H15	0.9300
C18—C11	1.479 (2)	C4—C3	1.375 (3)
C19—C20	1.385 (2)	C4—C5	1.382 (3)
C19—C24	1.390 (2)	C4—H4	0.9300
C1—C2	1.388 (2)	C5—H5	0.9300
C1—C10	1.489 (2)	C17—H17A	0.9600
C21—C20	1.379 (2)	C17—H17B	0.9600
C21—C22	1.380 (2)	C17—H17C	0.9600
C21—H21	0.9300	C3—H3	0.9300
C16—C15	1.347 (3)	C25—H25A	0.9600
C16—H16	0.9300	C25—H25B	0.9600
C12—C13	1.402 (2)	C25—H25C	0.9600
C12—C11	1.407 (2)		
			
C16—N—C8	129.76 (15)	C21—C20—C19	121.72 (15)
C16—N—C12	121.34 (15)	C21—C20—H20	119.1
C8—N—C12	108.89 (13)	C19—C20—H20	119.1
N—C8—C9	107.44 (14)	C14—C13—C12	120.95 (18)
N—C8—C7	126.80 (14)	C14—C13—H13	119.5
C9—C8—C7	125.71 (15)	C12—C13—H13	119.5
C8—C9—C11	109.24 (14)	O4—C22—C21	125.05 (16)
C8—C9—C10	119.70 (15)	O4—C22—C23	115.09 (16)
C11—C9—C10	130.71 (15)	C21—C22—C23	119.86 (16)
C5—C6—C1	119.22 (17)	C3—C2—C1	120.6 (2)
C5—C6—C7	119.38 (17)	C3—C2—H2	119.7
C1—C6—C7	121.39 (15)	C1—C2—H2	119.7
O3—C18—C19	120.77 (16)	C9—C11—C12	106.48 (14)
O3—C18—C11	120.06 (16)	C9—C11—C18	130.43 (15)
C19—C18—C11	119.03 (14)	C12—C11—C18	122.99 (15)
C20—C19—C24	117.97 (15)	C23—C24—C19	120.92 (16)
C20—C19—C18	122.49 (15)	C23—C24—H24	119.5
C24—C19—C18	119.50 (15)	C19—C24—H24	119.5
C2—C1—C6	119.25 (17)	C16—C15—C14	122.00 (17)
C2—C1—C10	119.19 (17)	C16—C15—H15	119.0
C6—C1—C10	121.54 (15)	C14—C15—H15	119.0
C20—C21—C22	119.14 (16)	C3—C4—C5	120.08 (19)
C20—C21—H21	120.4	C3—C4—H4	120.0
C22—C21—H21	120.4	C5—C4—H4	120.0
C15—C16—N	119.00 (18)	C4—C5—C6	120.7 (2)
C15—C16—H16	120.5	C4—C5—H5	119.6
N—C16—H16	120.5	C6—C5—H5	119.6
O1—C10—C9	122.39 (16)	C14—C17—H17A	109.5
O1—C10—C1	121.42 (16)	C14—C17—H17B	109.5
C9—C10—C1	116.13 (15)	H17A—C17—H17B	109.5
N—C12—C13	118.38 (15)	C14—C17—H17C	109.5
N—C12—C11	107.93 (14)	H17A—C17—H17C	109.5
C13—C12—C11	133.63 (17)	H17B—C17—H17C	109.5
O2—C7—C8	123.46 (17)	C4—C3—C2	120.1 (2)
O2—C7—C6	121.77 (16)	C4—C3—H3	119.9
C8—C7—C6	114.76 (15)	C2—C3—H3	119.9
C13—C14—C15	118.33 (17)	O4—C25—H25A	109.5
C13—C14—C17	121.7 (2)	O4—C25—H25B	109.5
C15—C14—C17	119.98 (18)	H25A—C25—H25B	109.5
C24—C23—C22	120.34 (17)	O4—C25—H25C	109.5
C24—C23—H23	119.8	H25A—C25—H25C	109.5
C22—C23—H23	119.8	H25B—C25—H25C	109.5
C22—O4—C25	118.39 (16)		
			
C16—N—C8—C9	−178.53 (15)	C22—C21—C20—C19	−1.8 (3)
C12—N—C8—C9	0.38 (17)	C24—C19—C20—C21	−0.2 (3)
C16—N—C8—C7	−1.0 (3)	C18—C19—C20—C21	−177.69 (16)
C12—N—C8—C7	177.96 (15)	C15—C14—C13—C12	−1.4 (3)
N—C8—C9—C11	0.40 (18)	C17—C14—C13—C12	177.84 (17)
C7—C8—C9—C11	−177.21 (15)	N—C12—C13—C14	0.7 (2)
N—C8—C9—C10	−173.46 (14)	C11—C12—C13—C14	−176.11 (17)
C7—C8—C9—C10	8.9 (2)	C25—O4—C22—C21	−2.3 (3)
O3—C18—C19—C20	154.62 (18)	C25—O4—C22—C23	178.04 (18)
C11—C18—C19—C20	−21.0 (2)	C20—C21—C22—O4	−177.84 (17)
O3—C18—C19—C24	−22.9 (3)	C20—C21—C22—C23	1.8 (3)
C11—C18—C19—C24	161.52 (16)	C24—C23—C22—O4	179.80 (17)
C5—C6—C1—C2	0.4 (3)	C24—C23—C22—C21	0.1 (3)
C7—C6—C1—C2	−178.47 (16)	C6—C1—C2—C3	−1.5 (3)
C5—C6—C1—C10	179.22 (16)	C10—C1—C2—C3	179.66 (17)
C7—C6—C1—C10	0.3 (2)	C8—C9—C11—C12	−1.00 (18)
C8—N—C16—C15	177.94 (16)	C10—C9—C11—C12	171.95 (17)
C12—N—C16—C15	−0.9 (2)	C8—C9—C11—C18	175.43 (16)
C8—C9—C10—O1	167.00 (17)	C10—C9—C11—C18	−11.6 (3)
C11—C9—C10—O1	−5.3 (3)	N—C12—C11—C9	1.22 (18)
C8—C9—C10—C1	−10.4 (2)	C13—C12—C11—C9	178.26 (17)
C11—C9—C10—C1	177.31 (16)	N—C12—C11—C18	−175.55 (14)
C2—C1—C10—O1	7.4 (3)	C13—C12—C11—C18	1.5 (3)
C6—C1—C10—O1	−171.35 (17)	O3—C18—C11—C9	140.6 (2)
C2—C1—C10—C9	−175.19 (16)	C19—C18—C11—C9	−43.8 (3)
C6—C1—C10—C9	6.0 (2)	O3—C18—C11—C12	−43.5 (3)
C16—N—C12—C13	0.5 (2)	C19—C18—C11—C12	132.13 (17)
C8—N—C12—C13	−178.57 (14)	C22—C23—C24—C19	−2.1 (3)
C16—N—C12—C11	178.02 (14)	C20—C19—C24—C23	2.1 (3)
C8—N—C12—C11	−1.00 (17)	C18—C19—C24—C23	179.74 (17)
N—C8—C7—O2	−0.2 (3)	N—C16—C15—C14	0.2 (3)
C9—C8—C7—O2	176.99 (17)	C13—C14—C15—C16	1.0 (3)
N—C8—C7—C6	−179.40 (14)	C17—C14—C15—C16	−178.26 (18)
C9—C8—C7—C6	−2.2 (2)	C3—C4—C5—C6	−0.7 (3)
C5—C6—C7—O2	−0.6 (3)	C1—C6—C5—C4	0.7 (3)
C1—C6—C7—O2	178.29 (16)	C7—C6—C5—C4	179.60 (16)
C5—C6—C7—C8	178.63 (15)	C5—C4—C3—C2	−0.4 (3)
C1—C6—C7—C8	−2.5 (2)	C1—C2—C3—C4	1.5 (3)
